# Non-coding RNAs as key regulators of epithelial-mesenchymal transition in breast cancer

**DOI:** 10.3389/fcell.2025.1544310

**Published:** 2025-03-25

**Authors:** Jing Peng, Wenhui Liu, Jiaju Tian, Yuncong Shu, Rui Zhao, Yuping Wang

**Affiliations:** ^1^ The First Clinical Medical College, Lanzhou University, Lanzhou, China; ^2^ School of life science, Lanzhou University, Lanzhou, China; ^3^ Department of Gastroenterology, The First Hospital of Lanzhou University, Lanzhou, China; ^4^ Gansu Province Clinical Research Center for Digestive Diseases, The First Hospital of Lanzhou University, Lanzhou, China

**Keywords:** breast cancer, epithelial-mesenchymal transition, noncoding RNAs, miRNAs, CircRNAs, lncRNAs, diagnostic biomarkers, therapeutic targets

## Abstract

This study examines the critical role of non-coding RNAs (ncRNAs) in regulating epithelial-mesenchymal transition (EMT) in breast cancer, a prevalent malignancy with significant metastatic potential. EMT, wherein cancer cells acquire mesenchymal traits, is fundamental to metastasis. ncRNAs—such as microRNAs (miRNAs), long non-coding RNAs (lncRNAs), and circular RNAs (circRNAs)—modulate EMT by influencing gene expression and signaling pathways, affecting cancer cell migration and invasion. This review consolidates recent findings on ncRNA-mediated EMT regulation and explores their diagnostic and therapeutic potential. Specifically, miRNAs inhibit EMT-related transcription factors, while lncRNAs and circRNAs regulate gene expression through interactions with miRNAs, impacting EMT progression. Given the influence of ncRNAs on metastasis and therapeutic resistance, advancing ncRNA-based biomarkers and treatments holds promise for improving breast cancer outcomes.

## 1 Introduction

Breast cancer (BC) ranks among the most prevalent malignancies globally as the second most common cancer worldwide (Bray et al., 2024). Over the past 3 decades, an upward trend in incidence has been observed. Breast, lung, and colorectal cancers collectively account for 51% of newly diagnosed malignancies, with breast cancer alone comprising 32% (Siegel et al., 2024). Among women, breast cancer is the most frequently diagnosed malignancy and a primary cause of cancer-related mortality. Approximately 25% of female cancer cases globally are attributed to breast cancer, which is responsible for nearly one-fourth of cancer deaths (Bray et al., 2024). Early detection through screening, heightened awareness, and treatment advances support an elevated 5-year survival rate for breast cancer, reaching 99% when the disease remains localized to the breast (Hushmandi et al., 2024). Current treatment options for breast cancer include surgery (e.g., lumpectomy or mastectomy), radiation therapy, chemotherapy, targeted therapy (e.g., HER2 inhibitors), and immunotherapy (e.g., immune checkpoint inhibitors). The choice of treatment depends on the cancer type, stage, and molecular characteristics. Non-coding RNAs (ncRNAs) play a critical role in cancer progression and metastasis. For example, in breast cancer, the upregulation of miR-21 has been associated with tumor growth and poor prognosis, while the downregulation of miR-145 is linked to increased invasion and metastasis (Table 1). However, the survival rate for metastatic breast cancer remains below 25% (Su et al., 2022). Breast tumors are histologically classified as either *in situ* or invasive carcinomas, further categorized into ductal or lobular subtypes. Ductal carcinoma *in situ* (DCIS) is the most common form of pre-invasive breast cancer. Although 10%–30% of DCIS cases may progress to invasive disease, biomarkers predicting progression to invasive or metastatic states remain limited (Nolan et al., 2023).

**TABLE 1:** Breast cancer subtypes: Classification, molecular features, and clinical management.

Breast cancer type	Morphologic feature	Molecular pattern	Diagnostic	Treatment	Preclinical/Clinical model result
Luminal A	Well-differentiated (Dawwas et al., 2019)	ER+ (SoRelle et al., 2020) PR+ (SoRelle et al., 2020) HER2- (Wolff et al., 2018)	Mammography (Siu and U.S. Preventive Services Task Force, 2016), biopsy (Waly et al., 2020)	Endocrine therapy (e.g.,,tamoxifen) (Tamoxifen for early breast cancer, 1998)	Improved survival in clinical trials (Pan et al., 2017)
Luminal B	Moderately differentiated (Dawwas et al., 2019)	ER+ (SoRelle et al., 2020) PR+ (SoRelle et al., 2020), HER2^+/−^(202)	Mammography (Siu and U.S. Preventive Services Task Force, 2016), biopsy (Waly et al., 2020)	Endocrine therapy + chemotherapy (Long et al., 2017)	Mixed response in preclinical models (Ades et al., 2014)
HER2-enriched	Poorly differentiated (Dawwas et al., 2019)	ER- (SoRelle et al., 2020) PR- (SoRelle et al., 2020) HER2+ (Slamon et al., 1987)	Immunohistochemistry (Wolff et al., 2013)	HER2-targeted therapy (e.g.,,trastuzumab) (Romond et al., 2005)	High efficacy in clinical trials (Piccart-Gebhart et al., 2005)
Triple-negative	Poorly differentiated (Dawwas et al., 2019)	ER- (SoRelle et al., 2020) PR- (SoRelle et al., 2020) HER2- (Wolff et al., 2018)	Biopsy (Waly et al., 2020), genetic testing (Akgün et al., 2019)	Chemotherapy (Eiermann and Vallis, 2012),immunotherapy (e.g., pembrolizumab) (Schmid et al., 2020)	Limited response in preclinical models (Lehmann et al., 2011)

Metastasis is the process by which a primary tumor develops secondary tumors at distant sites, marking advanced cancer progression and significantly affecting patient prognosis (Park et al., 2022). For most cancers, metastasis represents the terminal disease stage and involves complex cellular mechanisms, including detachment from the primary tumor, invasion, immune evasion, and modification of the tissue microenvironment. Many cancers rely on epithelial-mesenchymal transition (EMT), a critical event initiating cancer invasion and metastasis (Williams et al., 2019). Specifically, EMT, a process where malignant epithelial cells transform into mesenchymal cells, is considered the initial and essential step in malignancies’ invasion and metastasis, including breast cancer (Zhang H. et al., 2021). Given the limited therapeutic options and poor prognosis of metastatic breast cancer, identifying effective EMT-related molecular biomarkers is essential for improved diagnostics and therapeutic strategies.

Non-coding RNAs (ncRNAs) include microRNAs (miRNAs), long non-coding RNAs (lncRNAs), and circular RNAs (circRNAs). LncRNAs play key biological roles, such as regulating splicing, chromatin remodeling, translation, the cell cycle, gene imprinting, and mRNA decay (Lakshmi et al., 2021). Research has shown that dysregulated lncRNAs significantly impact survival and recurrence rates in breast cancer patients (Su et al., 2022). Additionally, miRNAs control gene expression by regulating mRNA and lncRNA degradation (Hashemipour et al., 2021). Emerging evidence links aberrant ncRNA expression to the development and progression of cancers, including breast cancer (Chen et al., 2021), liver cancer (Nagaraju et al., 2022), and lung cancer (Iqbal et al., 2019). Numerous studies indicate that most ncRNAs regulate invasion, migration, EMT, and metastasis, thus promoting breast cancer progression (Wu et al., 2019). These findings suggest ncRNAs as potential biomarkers for breast cancer diagnostics and as targets to improve treatment strategies.

This review consolidates current knowledge on ncRNAs, EMT-related ncRNAs, and their complex interactions with BC. It aims to deepen understanding of the biomarker potential of ncRNA-mediated EMT-regulatory pathways in BC and to encourage further investigation of their mechanistic role in EMT.

## 2 Breast cancer

BC, the most prevalent cancer among women worldwide, poses a significant global public health challenge (Feng et al., 2018). Since the mid-2000s, BC incidence has risen steadily by approximately 0.6% annually, as calculated from age-adjusted rates in population-based registries such as the Surveillance, Epidemiology, and End Results (SEER) program. During this period, the incidence of advanced disease increased by 0.7% per year, reflecting trends across all breast cancer subtypes rather than specific molecular alterations (e.g., Myc mutations) (DeSantis et al., 2019). BC encompasses a heterogeneous group of diseases with diverse biological and molecular origins, arising from regions within the breast, including ducts, lobules, or interstitial tissue (Feng et al., 2018). Most BCs are adenocarcinomas, with 85% originating from the ductal epithelium and 15% from the lobular epithelium. Ductal lesions range from ductal carcinoma *in situ* (DCIS) to invasive carcinoma, the latter spreading beyond the basement membrane into adjacent breast parenchyma. Other BC forms include Paget’s disease of the breast, inflammatory breast cancer, and rarer types, such as malignant phyllodes tumors and angiosarcomas (Katsura et al., 2022).

Breast cancer exhibits significant diversity in aggressiveness depending on the primary tumor site, with each subtype holding distinct prognostic and therapeutic implications. Clinically, breast tumors are categorized into three main groups based on the expression of estrogen receptor (ER), progesterone receptor (PR), and human epidermal growth factor receptor 2 (HER2/ERBB2): ER+, HER2+, and TNBC. ER + cancers constitute approximately 70% of all breast cancers. HER2+ tumors are further subdivided into HER2+ER+ (∼70%) and HER2+ER− (∼30%) subtypes, while TNBC (∼15% of cases) lacks ER, PR, and HER2 expression. TNBC frequently expresses epidermal growth factor receptor (EGFR) and cytokeratins CK5 and CK14, which are associated with basal-like features (Nolan et al., 2023). The distinction between HER2+ER+ and HER2+ER− subtypes is clinically significant due to their differing molecular profiles and treatment responses. HER2+ER + tumors often exhibit a less aggressive phenotype compared to HER2+ER− tumors, as the presence of ER signaling can modulate HER2-driven oncogenic pathways (Donovan et al., 2014). Additionally, HER2+ER + patients tend to have better overall survival and may benefit from combined HER2-targeted therapy (e.g., trastuzumab) and endocrine therapy (e.g., tamoxifen or aromatase inhibitors) (Denkert et al., 2017). In contrast, HER2+ER− tumors are typically more aggressive and rely heavily on HER2 signaling, making them more responsive to HER2-targeted therapies but less responsive to endocrine interventions (Donovan et al., 2014).

Breast cancer progression involves distinct stages, from localized tumors to invasive and metastatic disease, driven by molecular alterations such as the epithelial-mesenchymal transition (EMT). During EMT, key proteins like E-cadherin (epithelial marker) are downregulated, while N-cadherin and vimentin (mesenchymal markers) are upregulated, leading to loss of cell polarity and increased invasiveness (Thiery et al., 2009). Caveolin-1, a protein implicated in cell signaling and tumor suppression, has also been linked to EMT regulation in breast cancer (Cruz-Gonzalez et al., 2008). Additionally, non-coding RNAs (ncRNAs) play a pivotal role as mediators of EMT, influencing cancer cell plasticity and metastatic potential (Thomas et al., 2009).

Metastatic disease represents the most severe form of breast cancer, characterized by tumor spread from the breast to other body parts. TNBC, the most aggressive subtype, lacks ER, PR, and HER2 expression and accounts for 15%–20% of breast cancer cases. Often diagnosed at an advanced stage, TNBC has high recurrence rates and poor survival outcomes. Additionally, TNBC lacks effective targeted therapies and frequently metastasizes to the brain, bones, lungs, and liver (Park et al., 2022). Currently, up to 5% of patients present with incurable metastases, and an additional 10%–15% develop metastases within 3 years of diagnosis (McGuire et al., 2015).

Normal breast development and mammary stem cells are regulated by signaling pathways, including ER, HER2, and Wnt/β-catenin, which control stem cell proliferation, apoptosis, differentiation, and migration (Feng et al., 2018). In recent decades, gene analysis has become instrumental in characterizing cancer types and predicting treatment responses. Studies have identified the involvement of non-coding RNAs (ncRNAs) in various human diseases, including cancer (Iorio et al., 2008), with distinct miRNA types shown to play specific roles in the pathogenesis of breast and prostate cancers (Sidorova et al., 2023). For instance, members of the miR-324 and let-7 families act as tumor suppressors within cellular and tumor microenvironments in multiple cancers (Zhang et al., 2024). Overexpression of the miR-200 family can suppress metastasis by confining tumors to primary sites or promote metastasis by enhancing colonization at secondary sites (Dykxhoorn, 2010). Additionally, evidence indicates that epigenetic regulation and ncRNAs significantly contribute to cancer development and metastasis, especially by driving EMT in breast cancer (Wei et al., 2017; Thakur et al., 2024). Exploring ncRNAs’ roles in EMT regulation in breast cancer is crucial for identifying novel molecular targets to define BC phenotypes and predict clinical outcomes.

## 3 Application of EMT in breast cancer

EMT in breast cancer, involving the shift from an epithelial to a mesenchymal state, is extensively studied due to its critical role in tumor invasion and metastasis (Liu et al., 2015). The mesenchymal state is characterized by a loss of epithelial features, such as cell polarity and adhesion, and the acquisition of mesenchymal traits, including spindle-shaped morphology, increased motility, and expression of mesenchymal markers like N-cadherin and vimentin (Nieto et al., 2016). This transition enables cancer cells to detach from the primary tumor, invade surrounding tissues, and disseminate to distant sites, thereby driving metastasis (Lamouille et al., 2014). Metastasis and recurrence, the leading causes of mortality in breast cancer patients, are closely tied to EMT, which facilitates progression from *in situ* carcinoma to invasive cancer and is linked to drug resistance in tumor cells (Gulei et al., 2017). EMT is regulated by several transcription factors, including ZEB1/2, basic helix-loop-helix (bHLH) proteins like Twist, and the Snail family (Snail, Slug). Key signaling pathways, such as WNT, TGF-β, NOTCH, and Shh, activate EMT-related transcription factors like Snail, SLUG, ZEB1/2, and TWIST. Recent evidence suggests that interactions between microRNAs, lncRNAs, and EMT transcription factors are crucial in EMT regulation (Nieszporek et al., 2019).

Recent studies have updated the biomarker landscape for EMT and MET in breast cancer. Loss of E-cadherin expression in breast cancer is associated with decreased differentiation, increased invasiveness, higher tumor grade, metastatic potential, and poor prognosis (Liu F. et al., 2016). These biomarkers are essential for identifying tumor subtypes at greater risk of recurrence, metastasis, and treatment resistance, factors that contribute to mortality. Mechanistically, the TGF-β, Notch, and WNT signaling pathways can induce EMT through various mechanisms (Felipe Lima et al., 2016). Specifically, TGF-β induces EMT by binding to TβR-III and recruiting TβR-I, activating Smad proteins that regulate TGF-β-associated gene expression (Moustakas and Heldin, 2007). In breast cancer, TGF-β enhances tumor cell migration and invasion, driving EMT by modulating EMT markers such as E-cadherin, N-cadherin, and vimentin (Miettinen et al., 1994; Piek et al., 1999). Epithelial-mesenchymal transition is closely linked to breast cancer stem cell (CSC) properties, which are thought to drive tumor metastasis and recurrence (Reisenauer et al., 2021). EMT also increases breast cancer cell resistance to certain chemotherapeutic agents (Reisenauer et al., 2021). Key transcription factors, including Snail, Slug, Twist, and the ZEB family, regulate EMT by promoting tumor invasion and metastasis. They achieve this by suppressing epithelial markers, upregulating mesenchymal markers, reducing cell-cell adhesion, and increasing secretion of extracellular matrix-degrading enzymes (Banyard and Bielenberg, 2015). Additionally, loss of GATA3 function has been associated with EMT and tumor metastasis in breast cancer. GATA3 deficiency induces expression changes in EMT-related transcription factors, such as Fra1 and c-Fos, activating EMT and promoting breast cancer progression and metastasis (Liu et al., 2023).

**FIGURE 1:**
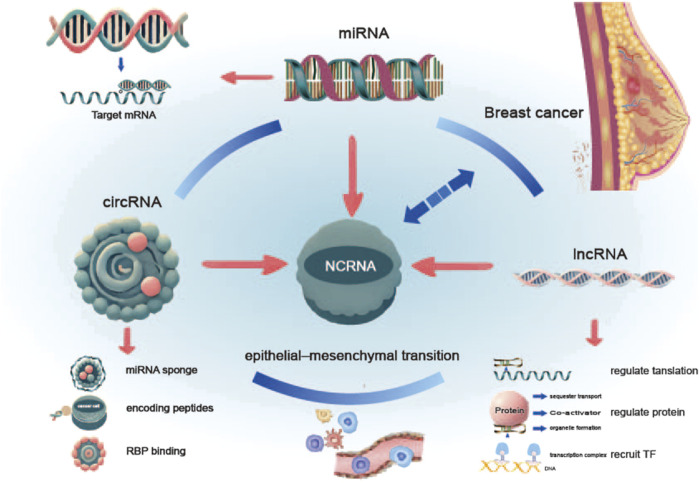
Role of ncRNAs in breast cancer and the EMT. The diagram illustrates the role of non-coding RNAs (ncRNAs) in breast cancer and the epithelial-mesenchymal transition (EMT). miRNAs, lncRNAs, and circRNAs regulate the initiation and progression of breast cancer through distinct mechanisms. These ncRNAs, via their complex regulatory networks, collectively promote the EMT process, thereby enhancing the invasiveness and metastatic potential of breast cancer cells.

These findings highlight the significance of EMT in breast cancer therapy and drug development, particularly in targeting key molecules and signaling pathways involved in the EMT process. Such research is essential for identifying novel therapeutic targets and improving breast cancer prognosis. Additionally, these studies have informed therapeutic strategies targeting CSCs. RNA, particularly ncRNA, is known to regulate development and differentiation and is involved in critical biological processes. Consequently, ncRNA-mediated gene regulation in EMT, metastasis, and apoptosis holds promise for advancing our understanding of cancer pathogenesis. Research has shown that alterations in miRNA expression significantly contribute to the development of most, if not all, human malignancies (Croce, 2009). In breast cancer, substantial research highlights the regulatory role of ncRNAs in EMT. For instance, studies indicate that silencing ZEB2NAT inhibits breast cancer cell proliferation, suggesting that ZEB2NAT may positively regulate cancer cell growth (Eroğlu Güneş et al., 2021). Future research could develop novel therapeutic approaches targeting ncRNA-regulated EMT pathways to improve breast cancer prognosis (Figure 1).

## 4 Non-coding RNAs

### 4.1 The role of key ncRNAs in regulating EMT in cancer

Launched in 2003 by the NHGRI, the ENCODE project aims to identify and annotate all functional elements in the human genome, including coding and non-coding regions. It has uncovered over 28,000 distinct lncRNAs, many still under investigation (The ENCODE Project Consortium, 2012). ENCODE’s comprehensive expression maps and functional networks have deepened our understanding of lncRNAs’ regulatory roles (Djebali et al., 2012). These insights highlight the non-coding genome’s potential as a source of cancer biomarkers and therapeutic targets (The ENCODE Project Consortium, 2011). Dysregulated lncRNAs drive tumor initiation, progression, and metastasis (Iyer et al., 2015). Recent transcriptomic analyses using next-generation sequencing have shown that thousands of ncRNAs exhibit abnormal expression or mutations in various cancers (Bhan et al., 2017). Although only about 1.22% of the human genome encodes proteins, over 80% is transcribed into ncRNAs, including circRNAs and lncRNAs (Consortium, 2012). NcRNAs are classified by length, morphology, and action site, with primary types like miRNAs, lncRNAs, circRNAs, and PIWI-interacting RNAs (piRNAs) playing diverse roles in cancer. Once overlooked, ncRNAs are recognized for their critical roles in cancer progression. For instance, certain miRNAs are highly expressed in cancer cells and facilitate tumor development, while others regulate multiple cancer types (Hou et al., 2014). MiRNAs, small RNAs approximately 22 nucleotides long, suppress protein translation or promote mRNA degradation by binding to the 3′untranslated region (3′UTR) of target mRNAs (Yu et al., 2014). lncRNAs and circRNAs are generally over 200 nucleotides long; however, lncRNAs are linear, while circRNAs have a circular structure (Palazzo and Lee, 2015). LncRNAs participate in various biological processes, including cell proliferation, differentiation, chromatin remodeling, epigenetic regulation, transcription, and post-transcriptional modification. Studies indicate that lncRNAs can promote breast cancer cell proliferation or inhibit apoptosis (Bin et al., 2018). CircRNAs, a large class of endogenous RNAs, are generated through exon skipping or back-splicing events. They regulate gene expression at multiple levels by binding to RNA-binding proteins (RBPs), sequestering endogenous miRNAs, or translating into proteins, thus playing critical roles in physiological functions (Hansen et al., 2013). The following sections will explore how specific non-coding RNAs impact cancer biology and their clinical implications.

### 4.2 Composition and functional roles of ncRNAs

#### 4.2.1 miRNAs

MicroRNAs are small non-coding RNAs, approximately 21–25 nucleotides long, that regulate gene expression by inhibiting degradation or translation of target mRNAs. Transcribed by RNA polymerase II as primary miRNAs (pri-miRNAs), miRNAs are processed in the nucleus by the Drosha-DGCR8 complex into precursor miRNAs (pre-miRNAs). Pre-miRNAs are then exported to the cytoplasm by Exportin-5 and cleaved into mature miRNAs by the Dicer protein (Lee et al., 2002). Mature miRNAs bind to the RNA-induced silencing complex (RISC) and interacting with Argonaute family proteins. Typically, miRNAs pair with complementary sequences in the 3′untranslated region (3′UTR) of target mRNAs, leading to mRNA degradation or translational repression and thus regulating gene expression (Filipowicz et al., 2008).

MiRNAs participate in various biological processes, including cell differentiation, proliferation, apoptosis, and tissue development. In breast cancer, abnormal miRNA expression is linked to tumor progression, metastasis, and therapeutic resistance. Specific miRNAs, such as the miR-200 family, inhibit breast cancer cell invasion and metastasis by suppressing EMT-related transcription factors ZEB1 and ZEB2 (Park et al., 2008). Additionally, miRNAs can indirectly influence tumor development by modulating immune cells and angiogenesis within the tumor microenvironment (Patil et al., 2020). MiRNA expression in exosomes varies across cancer types and stages, making miRNAs valuable biomarkers for cancer diagnosis and prognosis, though findings across studies remain inconsistent (Ono et al., 2015).

MiRNAs are key regulators of EMT, a process where cells lose epithelial characteristics and gain mesenchymal traits essential for tumor progression and metastasis. By targeting specific mRNA molecules, miRNAs control genes associated with EMT, affecting the synthesis of EMT-related proteins (McGuire et al., 2015). The miR-200 family members, including miR-200a, miR-200b, miR-200c, and miR-141, are known EMT inhibitors (Mongroo and Rustgi, 2010). Another miRNA, miR-205, is linked to improved prognosis in breast cancer patients as it reduces ZEB2 expression and limits EMT (Park et al., 2008). Additionally, the miR-30 family (e.g., miR-30a and miR-30d) inhibits EMT by targeting key regulators TWIST1 and SNAI1, with its downregulation in various cancers correlating with increased invasiveness and metastatic potential (Mao et al., 2018).

#### 4.2.2 LncRNAs

LncRNAs share several similarities with mRNA in transcription and processing (Wu et al., 2019). Like mRNA, lncRNAs are transcribed by RNA polymerase II from promoter regions associated with chromatin and undergo splicing, 5′capping, and 3′polyadenylation. These similarities suggest that lncRNAs follow transcriptional and processing pathways akin to mRNA. However, unlike mRNA, lncRNAs often show more specific expression patterns, likely due to complex regulatory mechanisms (Wu et al., 2019). Additionally, lncRNAs may contain sequence motifs that recruit nuclear factors, affecting their nuclear localization (Statello et al., 2021). Many lncRNAs are transported to the cytoplasm and have also been detected in exosomes in human blood. While the mechanisms of lncRNA sorting into exosomes remain unclear, it is speculated that specific lncRNA sequences interact with RNA-binding proteins (Statello et al., 2018). The distinct expression of lncRNAs in exosomes makes them valuable biomarkers for various diseases, including cancer.

As the potential of lncRNAs as stable serum biomarkers gains recognition, their application in cancer diagnostics is attracting significant attention (Qi et al., 2016). Unlike the relatively simple regulatory mechanisms of miRNAs, lncRNAs interact with DNA, RNA, and proteins through complex regulatory processes, including chromatin modification, transcriptional interference, and regulation of gene expression at post-transcriptional, translational, and post-translational levels (Lee et al., 2002). As critical components of the tumor microenvironment, exosomal lncRNAs play essential roles in cancer progression, mirroring the multifunctionality of exosomal miRNAs. These roles include promoting tumor cell proliferation, metastasis, angiogenesis, immune evasion, and drug resistance (Pathania and Challagundla, 2021; Zhang W. et al., 2021).

LncRNAs play a complex role in regulating EMT in breast cancer. They influence EMT through multiple mechanisms, including direct interactions with EMT-related transcription factors, affecting epigenetic modifications, serving as signaling molecules in intercellular communication, and modulating intracellular signaling pathways (Grelet et al., 2017). For instance, lncRNA ZEB2-AS1 promotes proliferation, metastasis, and EMT in triple-negative breast cancer by epigenetically activating ZEB2 (Zhang et al., 2019). Additionally, lncRNAs can act as miRNA “sponges,” sequestering miRNAs to reduce their suppression of target mRNAs, thus promoting tumor cell invasion and metastasis (Gregory et al., 2008a). LncRNAs also recruit histone-modifying enzymes to modify chromatin structure and regulate EMT-related gene expression (Liu S. J. et al., 2021). Furthermore, lncRNAs are transmitted via exosomes, providing a mechanism for intercellular communication that influences EMT in recipient cells (Melo et al., 2014). LncRNAs can also impact miRNA maturation by modulating miRNA-processing enzyme activity, thereby affecting EMT (Huarte et al., 2010). Lastly, lncRNAs regulate intracellular signaling pathways, such as MAPK/ERK and PI3K/AKT, influencing EMT, tumor cell proliferation, and survival (Zhou et al., 2018).

#### 4.2.3 circRNAs

CircRNAs are a class of non-coding RNAs with a covalently closed loop structure, lacking 5′–3′ polarity and a polyadenylated tail (Liu et al., 2018). This structure confers remarkable stability, rendering circRNAs resistant to degradation by RNA exonucleases (Memczak et al., 2013). CircRNA biogenesis begins in the nucleus, where RNA polymerase II transcribes circRNAs from promoter regions associated with chromatin, forming circular molecules with distinct functions. CircRNAs regulate gene expression through various mechanisms, including acting as miRNA sponges, modulating transcription factor activity, and participating in cellular signaling pathways (Patop and Kadener, 2018). For example, circ-Ccnb1 induces breast cancer cell death by interacting with p53 via H2AX in wild-type p53 cells and with Bclaf1 via H2AX in p53-mutant cells (Shang et al., 2019).

CircRNAs have garnered attention for their role in breast cancer onset and progression, particularly in cell proliferation and EMT (Shang et al., 2019). CircRNAs typically regulate gene expression by modulating miRNA activity (Tian et al., 2022). By interacting with miRNAs, circRNAs diminish miRNA inhibition of EMT-related genes, thus facilitating EMT. For example, circRNAs act as miRNA “sponges,” thereby driving EMT (Fan et al., 2021). Beyond this sponge effect, circRNAs can interact directly with transcription factors to regulate EMT-related genes or influence EMT through pathways such as TGF-β and Wnt/β-catenin (Qu et al., 2015; Zhao et al., 2013). As components of exosomes, circRNAs also facilitate intercellular communication, transmitting signals to nearby or distant cells, impacting EMT and modifying the tumor microenvironment (Liu J. et al., 2021). Abnormal circRNA expression in breast cancer tissue is associated with tumor invasiveness, metastatic potential, and patient prognosis. For instance, circ_RPPH1 promotes breast cancer progression by sponging miR-542-3p and upregulating ARHGAP1 (Qi et al., 2022). Thus, circRNAs are crucial regulators of EMT in breast cancer, offering potential biomarkers and therapeutic targets.

### 4.3 Dysregulation of ncRNAs in breast cancer

Research on ncRNAs in breast cancer has significantly advanced, highlighting their essential roles in disease progression (Grelet et al., 2017). Specific ncRNA expression levels in breast cancer tissues are closely linked to tumor aggressiveness, metastatic potential, and patient prognosis. For instance, lncRNA HOTAIR promotes breast cancer cell invasion and metastasis by interacting with the polycomb repressive complex 2 (PRC2) and lysine-specific demethylase 1 (LSD1), contributing to gene silencing and chromatin remodeling (Coan et al., 2024). Clinically, dysregulated lncRNAs are correlated with various clinicopathological characteristics, including molecular subtypes, tumor grade, lymph node metastasis, and distant metastasis (Meng et al., 2024). For example, lncRNA-PTENP1 is downregulated in breast cancer tissues and negatively correlated with higher tumor stages, whereas lncRNA-RACGAP1P is overexpressed and positively associated with lymph node and distant metastasis, as well as TNM staging (Gupta et al., 2024).

**FIGURE 2:**
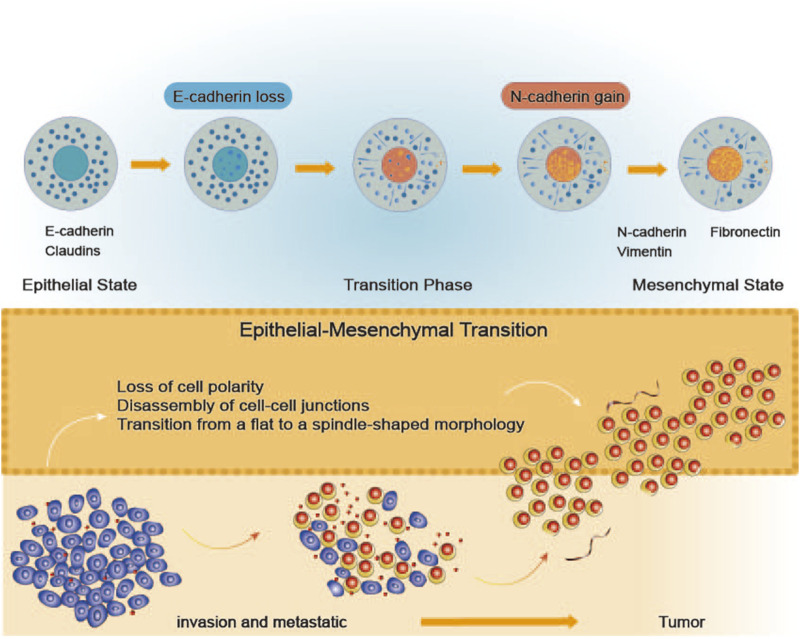
The Process of Epithelial-Mesenchymal Transition. The diagram depicts the process of epithelial-mesenchymal transition (EMT), during which cells gradually shift from an epithelial state to a mesenchymal state. In the epithelial state, cells express E-cadherin and claudins, which are crucial for maintaining intercellular adhesion and polarity. As the transition progresses, cells progressively lose E-cadherin and assume a mesenchymal phenotype, characterized by the expression of N-cadherin, vimentin, and fibronectin. Throughout this process, cells lose their polarity, cell junctions disassemble, and the cell morphology changes from a flat to a spindle-like shape. Ultimately, these changes enhance cellular motility, resulting in increased invasiveness and metastatic potential.

NcRNAs also show potential in early breast cancer screening and diagnosis. For example, blood-based miRNA expression profiles could serve as biomarkers for early breast cancer detection (Yan and Bu, 2021). Additionally, the expression of lncRNA MALAT1 is linked to tumor aggressiveness and poor prognosis in breast cancer, presenting a novel molecular target for diagnosis and therapy (Coan et al., 2024). Prognostically, certain lncRNA levels may predict breast cancer survival outcomes. High TROJAN expression has been detected in breast cancer tissues and is positively correlated with tumor size and pathological grade (Jin et al., 2020). Beyond the lack of reliable biomarkers, limited therapeutic options contribute to breast cancer-related mortality. Thus, identifying novel molecular markers for diagnosis, treatment, and prognosis is critical. The role of ncRNAs in EMT is especially significant, as ncRNAs are closely associated with clinical outcomes in breast cancer and hold promise as diagnostic biomarkers and therapeutic targets for future treatment (Figure 2).

## 5 ncRNAs and EMT in breast cancer

Research has shown that ncRNAs play a crucial role in regulating EMT and MET in breast cancer, influencing tumor progression and metastasis (Buyuk et al., 2022). NcRNAs modulate these processes through various mechanisms, effectively targeting and influencing one or multiple steps in these regulatory pathways. They do so by (i) directly inhibiting one or more EMT transcription factors (EMT-TFs) or by regulating cytoskeletal components (epithelial and mesenchymal genes) (Wang H. B. et al., 2019; Ma et al., 2020), or (ii) by modulating key signaling pathways involved in EMT (Sun et al., 2019; Wang J. et al., 2019). Studies indicate that specific ncRNA expression patterns just correlate with clinicopathological features such as molecular subtypes, tumor grade, lymph node metastasis, and distant metastasis in breast cancer (Sarrió et al., 2008). NcRNA regulation often operates via bidirectional feedback loops, enabling efficient control of EMT. The following sections will focus on studies examining the roles of oncogenic and tumor-suppressive ncRNAs in EMT regulation in breast cancer. Understanding ncRNA-mediated EMT may elucidate mechanisms of breast cancer progression and metastasis and identify novel therapeutic targets.

**FIGURE 3:**
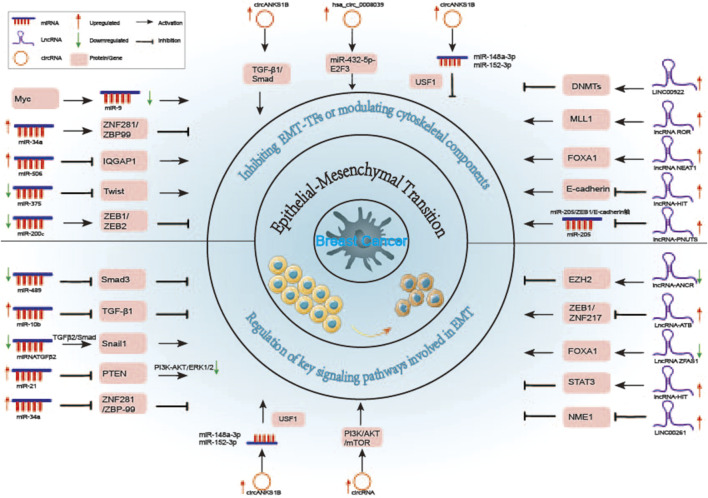
Non-Coding RNA Networks Regulating Epithelial-Mesenchymal Transition in Breast Cancer. The role and molecular mechanisms of long non-coding RNAs (lncRNAs) in the epithelial-mesenchymal transition (EMT) of breast cancer have been extensively studied. Specific lncRNAs have been shown to modulate EMT by positively or negatively regulating key target genes and signaling pathways, thereby contributing to the progression of breast cancer. These lncRNAs influence various aspects of tumor cell behavior, including migration, invasion, and metastasis, highlighting their critical involvement in cancer development.

Several tumor-suppressive ncRNAs inhibit breast cancer EMT by directly repressing EMT transcription factors (EMT-TFs) or targeting signaling pathway components that regulate EMT. Modulating key EMT-related pathways is a common mechanism through which these ncRNAs exert their effects (Gonzalez and Medici, 2014). Understanding the regulatory roles of these ncRNAs in EMT could inform novel therapeutic strategies to prevent or reduce metastasis (Figure 3; Table 2).

**TABLE 2:** NcRNAs functioning in the EMT of BC.

ncRNA	Target or the whole pathway	EMT	References
miR-200c	ZEB1 ZEB2	(−)	Feng et al. (2014)
miR-375	Twist	(−)	Yu et al. (2014)
miR-506	IQGAP1	(−)	Sun et al. (2015a)
miR-34a	ZNF281/ZBP99	(−)	Mauhin et al. (1993)
miR-9	E-cadherin	(−)	Chen et al. (2019a), Khew-Goodall and Goodall (2010)
miR-125b	Sema4C	(−)	Zhou et al. (2013)
miR-182	SMAD7	(+)	Yu et al. (2016)
miR-10b	TGF-β1	(+)	Han et al. (2014)
miRNATGβ2 (miR-145、miR-200a、miR-141)	TGFβ2-Snail1-miRNA (TGFβ2)	(−)	Luo et al. (2024), Davenport et al. (2022)
miR-21	AKT/ERK1/2 PTEN	(−)	Han et al. (2012)
miR-34a	ZNF281/ZBP-99	(+)	Hahn et al. (2013)
miR-489	Smad3	(−)	Wang et al. (2010)
miR-888	E-cadherin	(+)	He et al. (2023)
miR-7	SETDB1	(−)	Zhang et al. (2014)
miR-381	CXCR4	(−)	Xue et al. (2017)
lncRNA-HIT	E-cadherin	(+)	Richards et al. (2015)
lncRNA-PNUTS	miR-205	(+)	Grelet et al. (2017)
lncRNA NEAT1	FOXA1	(+)	Liu et al. (2020)
lncRNA ROR	MLL1	(+)	Hu et al. (2021)
LINC00922	NKD2	(+)	Wang et al. (2021a)
lncRNA-ANCR	EZH2	(−)	Li et al. (2017a)
LncRNA-ATB	miR-200c	(+)	Yuan et al. (2014)
LncRNA ZFAS1	STAT3	(−)	Sharma et al. (2021)
LINC00261	NME1	(−)	Guo et al. (2020)
NEAT1	miR-410-3p/CCND1	(+)	Park et al. (2021)
circANKS1B	TGF-β1/Smad USF1	(+)	Zeng et al. (2018)
hsa_circ_0008039	miR-432-5p	(+)	Liu et al. (2018)

+means promoting, - means inhibiting.

### 5.1 Key MicroRNAs regulating EMT

MicroRNAs, such as the miR-200 family, miR-375, and miR-506, can target and inhibit EMT transcription factors, thereby promoting epithelial characteristics and suppressing the EMT process (Yu et al., 2014; Gregory et al., 2008b). When these ncRNAs are dysregulated, the loss of key microRNAs can be particularly consequential, potentially leading to the induction of EMT. The following examples illustrate this phenomenon.

#### 5.1.1 Direct inhibition of EMT-TFs or regulation of cytoskeletal components (epithelial and mesenchymal genes)

MiRNAs significantly influence chemoresistance in cancer cells, with the miR-200 family, especially miR-200c, being well-studied due to its links to chemoresistance and EMT (Feng et al., 2014). miR-200c and miR-200a are crucial for suppressing EMT by regulating epithelial markers like E-cadherin (Feng et al., 2014). They achieve this by targeting ZEB1 and ZEB2, transcriptional repressors of E-cadherin, thus preserving epithelial traits and inhibiting EMT (Bai et al., 2014). The miR-200 family binds to the 3′UTRs of ZEB1 and ZEB2, reducing their expression. Since ZEB1 and ZEB2 negatively regulate E-cadherin, their inhibition increases E-cadherin levels, supporting epithelial characteristics. E-cadherin, essential for cell adhesion, is generally downregulated during EMT, a process associated with tumor invasiveness and metastasis. Thus, miR-200 family regulation is crucial for preventing breast cancer cell invasion and metastasis (Stinson et al., 2011; Bojmar et al., 2013).

During the progression of epithelial cancers, including breast cancer, microRNA miR-375 regulates key processes by targeting Twist mRNA to inhibit translation and regulate EMT. Twist, a key transcription factor, promotes EMT and metastasis. By binding to Twist’s 3′UTR, miR-375 reduces Twist expression, slowing EMT (Yu et al., 2014). Twist inhibition by miR-375 also affects EMT-related molecules, including cell cycle proteins and extracellular matrix enzymes. These changes reduce cancer cell invasiveness and metastasis. Additionally, miR-375s regulation of Twist may influence the tumor microenvironment, affecting fibroblasts, immune cells, and angiogenesis. This modulation may further suppress tumor progression and metastasis.

MiR-506 curtails breast cancer cell invasion and metastasis by targeting IQGAP1 (IQ motif-containing GTPase-activating protein 1), an effector protein critical to cell migration and tumor invasiveness. By binding to the 3′UTR of IQGAP1 mRNA, miR-506 induces degradation or suppresses translation, reducing IQGAP1 protein levels. IQGAP1 downregulation weakens cancer cell migration and invasion. MiR-506 may also affect invasion-related signaling, including cytoskeletal reorganization and cell adhesion. Through these mechanisms, miR-506 likely inhibits EMT, a crucial step in tumor metastasis (Sun G. et al., 2015).

MiR-34a, often downregulated in tumors, suppresses breast cancer cell invasion and metastasis by targeting ZNF281. The transcriptional repressor Snail binds to the miR-34a promoter, decreasing its expression (Mauhin et al., 1993). Reduced miR-34a expression upregulates target genes like ZNF281, linked to cell cycle regulation and tumor cell migration. Thus, low miR-34a levels are associated with increased EMT and metastatic potential in breast cancer.

Additionally, Snail and miR-34a establish a positive feedback loop: Snail represses miR-34a, which regulates SNAIL via ZNF281. This regulatory loop amplifies breast cancer cell invasiveness and metastasis (Gebeshuber et al., 2009).

Myc, a transcription factor and oncogene, directly regulates miR-9 expression by binding to its promoter (Chen et al., 2019a). Elevated miR-9 expression is linked to EMT in breast cancer, targeting the 3′UTR of E-cadherin to suppress its expression. Reduced E-cadherin, a hallmark of EMT, promotes the loss of epithelial traits. By inhibiting E-cadherin, miR-9 fosters an invasive phenotype in breast cancer and is associated with tumor metastasis. MiR-9 likely facilitates metastasis by regulating genes involved in cell cycle, motility, and extracellular matrix remodeling (Khew-Goodall and Goodall, 2010).

MiR-125b is downregulated in chemoresistant breast cancer, and ectopic expression reverses the EMT phenotype. MiR-125b regulates EMT by targeting Sema4C, and its overexpression or Sema4C depletion resensitizes cells to docetaxel (Yang et al., 2015). Specifically, Downregulated miR-125b is closely associated with EMT activation in chemoresistant cells (Wang et al., 2013). MiR-125b mimics reduce migration and invasion in resistant cells and convert mesenchymal-like cells to an epithelial morphology. This effect is mediated, in part, through miR-125b′s targeting of Sema4C, whose reduced expression suppresses EMT (Zhou et al., 2013). These findings link miR-125b and Sema4C to chemoresistance by influencing cancer stem cell traits, suggesting they may be novel targets to reverse breast cancer chemoresistance (Wang et al., 2013).

In breast cancer, miR-182 promotes TGF-β-induced EMT and metastasis by targeting SMAD7. The TGF-β pathway regulates EMT and metastasis, with SMAD7 as a negative regulator that limits TGF-β effects (Heldin et al., 2012). However, studies have shown that in certain cancer cells, TGF-β induces SMAD7 transcription without raising protein levels, indicating post-transcriptional suppression by miR-182 (Yu et al., 2016; Massagué and Chen, 2000). TGF-β activates miR-182, which targets the 3′UTR of SMAD7 mRNA, suppressing translation and lifting inhibitory feedback on TGF-β, thereby amplifying EMT and invasion. Silencing miR-182 upregulates SMAD7, preventing TGF-β-induced EMT and invasion. Conversely, miR-182 overexpression promotes cancer cell invasion and TGF-β-induced osteoclast formation, aiding bone metastasis (Yu et al., 2016; Lei et al., 2014).

Additionally, MiR-182 expression inversely correlates with SMAD7 protein levels in tumor samples, suggesting a mechanism by which cancer cells evade TGF-β self-inhibition to promote metastasis. The miR-182/SMAD7/TGF-β axis thus represents a potential therapeutic target in breast cancer metastasis (Yu et al., 2016).

#### 5.1.2 Regulation of key signaling pathways involved in EMT

MiR-10b is pivotal in breast cancer EMT, a key step in metastasis. EMT involves loss of cell adhesion and polarity, promoting cell migration and invasion (Yang and Weinberg, 2008). MiR-10b upregulation in breast cancer correlates with increased aggressiveness (Zhang and Ma, 2012). Inhibition of miR-10b elevates E-cadherin and reduces vimentin, attenuating the EMT phenotype.

Transforming growth factor-beta (TGF-β) induces EMT across cell types, with TGF-β1 specifically targeting miR-10b (Han et al., 2014). In TGF-β1-induced EMT in breast cancer, miR-10b upregulation promotes EMT, invasion, and proliferation, effects that miR-10b inhibition can partially reverse. These findings highlight miR-10b′s role in TGF-β1-mediated EMT in breast cancer (Han et al., 2014).

The TGFβ2-Snail1-MiRNA circuit drives tumor invasion and metastasis. TGFβ2, predominantly overexpressed in TNBC, is crucial for maintaining mesenchymal and invasive traits (Luo et al., 2024). Activation of the TGFβ2/Smad signaling pathway upregulates the expression of Snail1, a key EMT transcription factor. Snail1 recruits EZH2 to silence miRNAs (such as miR-145, miR-200a, and miR-141) that suppress TGFβ2 (Kaufhold and Bonavida, 2014). This TGFβ2-Snail1/EZH2-miRNA loop functions specifically in TNBC, while in luminal subtypes, Estrogen receptor alpha (ERα) disrupts this loop by inhibiting Snail1. MiRNATGFβ2 is downregulated in TNBC and inversely correlates with TGFβ2 levels; its overexpression reverses TGFβ2-induced mesenchymal traits, reduces invasiveness, and enhances chemosensitivity. These findings underscore the therapeutic potential of targeting the TGFβ2-Snail1-miRNA circuit in TNBC and provide novel insights into EMT regulation (Kaufhold and Bonavida, 2014).

MiR-21 is essential for breast cancer invasion and metastasis, modulating EMT and CSC traits. hsa-miR-21 antagomir transfection in MDA-MB-231 cells downregulates miR-21, reversing EMT and CSC phenotypes. Mechanistically, inhibiting miR-21 upregulates PTEN, inactivating the AKT/ERK1/2 pathways (Han et al., 2012). The downregulation of miR-21 increases E-cadherin and decreases mesenchymal markers (N-cadherin, vimentin, α-SMA), significantly reducing cell migration and invasion (Liu M. H. et al., 2016). In addition, miR-21 inhibition reduces CSC marker expression (ALDH1+, CD44+/CD24−/low) and mammosphere formation, indicating diminished CSC traits. Mammospheres are three-dimensional cell clusters that form *in vitro* under non-adherent culture conditions and are enriched for breast cancer stem cells (CSCs) with self-renewal and tumor-initiating capabilities (Dontu et al., 2003). The ability to form mammospheres is a key functional assay for assessing CSC properties, as it reflects the cells’ capacity for clonal expansion and survival in anchorage-independent conditions (Dontu et al., 2003). Mechanistically, miR-21 targets PTEN’s 3′UTR, suppressing its expression. PTEN negatively regulates PI3K-AKT and MAPK/ERK1/2 pathways; miR-21 antagonism upregulates PTEN, reducing AKT and ERK1/2 phosphorylation and suppressing EMT and CSC traits. PI3K-AKT (LY294002) and ERK1/2 (U0126) inhibitors replicate miR-21 inhibition effects, confirming pathway roles in EMT and CSC regulation (Han et al., 2012; Liu M. H. et al., 2016). Regulating miR-21 expression offers a novel perspective and potential therapeutic target for modulating EMT and CSC properties in breast cancer.

ZNF281/ZBP-99 is a transcription factor with four Krüppel-type zinc fingers (Lisowsky et al., 1999). Snail, a major EMT transcription factor, activates ZNF281 transcription while repressing miR-34a/b/c expression (Kim et al., 2011; Siemens et al., 2011). MiR-34a/b/c microRNAs reduce ZNF281 mRNA, but SNAIL represses miR-34a to stabilize ZNF281 (Siemens et al., 2011). Activated p53 induces miR-34a, which suppresses ZNF281 in a p53-dependent manner. ZNF281 overexpression in colorectal cancer induces EMT, enhancing migration, invasion, and β-catenin activity (Toyota et al., 2008). It also promotes stem cell markers (LGR5, CD133) and spheroid formation. ZNF281 downregulation triggers MET, reducing migration, invasion, spheroids, and lung metastasis in mice. Additionally, c-MYC overexpression induces ZNF281 via SNAIL; ZNF281 inactivation prevents c-MYC- or SNAIL-driven EMT. ZNF281 expression increases with colorectal cancer progression and correlates with recurrence. As part of the EMT regulatory network, ZNF281 promotes breast cancer invasion and metastasis via interactions with miR-34a and SNAIL, highlighting a potential therapeutic target (Hahn et al., 2013).

MiRNAs are crucial in regulating chemoresistance in cancer therapy (Wang et al., 2010). In breast cancer treatment, the expression level of miR-489 has been shown to significantly influence chemosensitivity (Jiang et al., 2014). miR-489 targets Smad3 to inhibit EMT, reducing breast cancer invasion and metastasis. Smad3 is elevated in chemoresistant cells, but miR-489 overexpression downregulates it (Ahn et al., 2012), This miR-489–Smad3–EMT pathway has been confirmed in chemoresistant xenografts and clinical samples, positioning miR-489 as a promising therapeutic approach for resistant breast cancer (Wang et al., 2010). miR-489 suppresses EMT, invasion, and metastasis by targeting the Smad3-PI3K/Akt pathway. SPIN1, directly regulated by miR-489, is highly expressed in chemoresistant breast cancer tissues and inversely correlated with miR-489 levels. Elevated SPIN1 is associated with higher tumor grade, lymph node metastasis, advanced stage, and progesterone receptor (PR)-positive status (Dang et al., 2011). SPIN1 overexpression promotes cell migration, invasion, and inhibits apoptosis, counteracting miR-489s tumor-suppressive effects (Vincent et al., 2009). Key PI3K/Akt pathway molecules (PIK3CA, AKT, CREB1, BCL2) are overexpressed in chemoresistant tissue and act downstream of SPIN1. SPIN1 inhibition or miR-489 upregulation suppresses the PI3K/Akt pathway, suggesting a potential therapeutic approach to enhance chemosensitivity and reduce metastasis (Wang et al., 2010).

In breast cancer, miR-888 expression is linked to tumor invasiveness and metastasis (Sedaghati Burkhani and Salimi, 2022). Huang et al. demonstrated that miR-888 directly targets E-cadherin in MCF-7 side population (SP) cells, reducing E-cadherin mRNA and membrane-bound protein, thus weakening adhesion and enhancing metastatic potential (Batlle et al., 2000). miR-888 upregulation is associated with CSC-like traits in SP cells, including high metastasis and tumor formation capacity (Huang et al., 2014). E-cadherin dysregulation critically contributes to breast cancer metastasis and correlates with poor prognosis and survival. Loss of E-cadherin function arises from CDH1 mutations, chromosomal loss of heterozygosity, CDH1 promoter hypermethylation, transcriptional repression, and post-translational changes (Corso et al., 2020). CircRAD54L2 facilitates breast cancer progression by modulating the miR-888/PDK1 axis, acting as a ceRNA for miR-888 to relieve its inhibition of PDK1, thereby enhancing TNBC invasion and metastasis (He et al., 2023).

In breast cancer, lncRNA HOTAIR suppresses miR-7, influencing EMT and promoting invasion and metastasis (Okuda et al., 2013). Research by Zhang et al. demonstrated that HOTAIR downregulates miR-7 in breast cancer stem cells, while miR-7 directly targets SETDB1 to reverse EMT via the STAT3 pathway (Zhang et al., 2014). HOTAIR, an oncogenic lncRNA overexpressed in breast cancer, drives tumor progression by recruiting chromatin-modifying enzymes (Gupta et al., 2010). miR-7 acts as a tumor suppressor, with its low expression linked to poor prognosis (Foekens et al., 2008). miR-7 inhibits SETDB1, a histone methyltransferase associated with invasion, and blocks STAT3 activation, further suppressing EMT and metastasis (Zhang et al., 2014; Hassanie et al., 2024; Marotta et al., 2011).

MicroRNA miR-381 plays an inhibitory role in cancer cell proliferation, EMT, and metastasis through the precise regulation of specific molecular pathways. Research by Xue et al. uncovered that miR-381 suppresses breast cancer cell proliferation, EMT, and metastasis by targeting CXCR4. CXCR4 is a G protein-coupled receptor whose high expression is closely associated with tumor invasion and metastasis in several cancers, including breast cancer (Yang et al., 2014), pancreatic cancer (Sun J. S. et al., 2015), and prostate cancer (Akashi et al., 2008). MiR-381 directly binds to the 3′untranslated region (3′UTR) of CXCR4, inhibiting its expression and thereby blocking CXCR4-mediated signaling. This inhibition of CXCR4 expression leads to reduced breast cancer cell proliferation, decreased invasive capacity, and inhibition of the epithelial-to-mesenchymal transition (EMT) process (Xue et al., 2017). In breast cancer, miR-381 downregulation correlates with greater invasiveness, while its overexpression restores chemosensitivity and promotes drug-induced apoptosis by inhibiting FYN, a MAPK pathway component (Xue et al., 2017). Additionally, miR-221/222 has been shown to inhibit EMT under certain conditions, although some studies suggest that they may also promote EMT depending on the context (Nassirpour et al., 2013).

### 5.2 Key lncRNAs regulating EMT

Several lncRNAs have been identified as key regulators in the progression of EMT in breast cancer through various mechanisms. These lncRNAs influence critical aspects of tumor cell migration, invasion, and metastasis. The following outlines the major mechanisms by which these lncRNAs contribute to EMT in breast cancer.

#### 5.2.1 Direct inhibition of EMT transcription factors (EMT-TFs) or regulation of cytoskeletal components (epithelial and mesenchymal genes)

In breast cancer, the regulatory network of lncRNAs and miRNAs plays a crucial role in the EMT process, which is closely linked to cancer cell invasion and metastasis (Venkatesh et al., 2021). Richards et al. elucidated the involvement of lncRNAs in the TGF-β signaling pathway, particularly in the context of breast cancer. lncRNA-HIT promotes EMT by directly suppressing the expression of E-cadherin. Upon stimulation by TGF-β, the expression of lncRNA-HIT is upregulated, leading to the inhibition of E-cadherin, a critical event in the EMT process. The downregulation of E-cadherin weakens intercellular adhesion, enhancing the migratory and invasive capabilities of breast cancer cells, facilitating tumor dissemination and metastasis (Richards et al., 2015). LncRNA-HIT is significantly overexpressed in highly metastatic 4T1 breast cancer cells. Knockdown of lncRNA-HIT markedly reduces 4TI migratory and invasive potential (Richards et al., 2015). The TGF-β signaling pathway is a central regulatory factor in the EMT process in breast cancer, activating various downstream effectors through Smad-dependent and independent pathways, thereby promoting cancer cell invasion and metastasis (Nawshad et al., 2005; Javelaud and Mauviel, 2005). In this process, lncRNAs interact with miRNAs, affecting their stability and function, thereby regulating the expression of EMT-related genes. For instance, lncRNA HOTAIR functions as a molecular “sponge” for miR-129-5p, modulating the miR-129-5p/FZD7 axis, and promoting the proliferation, migration, and invasion of breast cancer cells (Wu et al., 2021). Moreover, the expression levels of lncRNAs are closely associated with the aggressiveness and prognosis of breast cancer. HOTAIR is highly expressed in various human cancers and is associated with poor prognosis, influencing tumor progression and therapeutic response by recruiting chromatin-modifying factors to regulate gene expression (Xin et al., 2021).

In breast cancer, lncRNA-PNUTS significantly influences EMT and tumor progression by facilitating an mRNA-to-lncRNA splicing mechanism (Dhamija and Diederichs, 2016). Grelet et al. revealed that lncRNA-PNUTS functions in EMT as a competitive sponge for miR-205 (Grelet et al., 2017). This action is regulated by hnRNP E1, which binds to an RNA structure within PNUTS precursor RNA to control its splicing (Chaudhury et al., 2010; Hussey et al., 2011). When hnRNP E1 is released—through silencing, nuclear export, or TGF-β signaling—alternative splicing produces the non-coding RNA isoform of PNUTS. lncRNA-PNUTS, highly expressed in mesenchymal-like breast cancer cells and tumor tissues, binds miR-205 to prevent it from inhibiting ZEB1 mRNA, promoting EMT. ZEB1, a transcriptional repressor, downregulates E-cadherin, enhancing breast cancer cell invasion and metastasis (Hussey et al., 2011). Thus, lncRNA-PNUTS critically regulates EMT and tumor progression in breast cancer by modulating the miR-205/ZEB1/E-cadherin axis.

In the progression of breast cancer, long non-coding RNA NEAT1 plays a pivotal role in promoting cancer cell proliferation and metastasis through multiple mechanisms. Research by Zhang et al. demonstrated that lncRNA NEAT1 enhances the invasive and metastatic potential of breast cancer cells by facilitating the EMT process (Zhang M. et al., 2017). NEAT1 functions as a “sponge” for microRNAs, particularly through its interaction with miR-23a-3p, which regulates the expression of FOXA1, thereby promoting cell proliferation and chemoresistance in breast cancer cells (Liu et al., 2020). Additionally, NEAT1 also accelerates progression via the miR-410-3p/CCND1 axis, where CCND1 (Cyclin D1) overexpression correlates with poor prognosis. The overexpression of CCND1 in breast cancer is strongly associated with poor prognosis (Shin et al., 2019). High NEAT1 expression is linked to tumor aggressiveness and therapeutic resistance; it interacts with miR-486-5p to regulate SMAD4, activating TGF-β/SMAD signaling essential for EMT and cancer stem cell traits (Wang and Zheng, 2020). NEAT1 is also crucial in breast cancer stem cell self-renewal and multidrug resistance. Targeting NEAT1 could enhance chemotherapy efficacy, as NEAT1 inhibition—such as through small interfering RNA (siRNA) —reduces proliferation and invasion while increasing drug sensitivity (Shin et al., 2019). In summary, NEAT1’s regulation of multiple pathways highlights its potential as a therapeutic target, providing new avenues for breast cancer treatment.

The overexpression of lncRNA ROR in breast cancer is significantly associated with tumor aggressiveness and poor prognosis. LncRNA ROR promotes EMT in breast cancer cells by recruiting histone methyltransferase MLL1, which enhances TIMP3 transcription through H3K4 trimethylation at its promoter region (Hu et al., 2021). TIMP3, a tissue inhibitor of metalloproteinases, is upregulated in breast cancer, correlating strongly with EMT and tumor invasiveness (Hu et al., 2021). Consequently, lncRNA ROR facilitates breast cancer cell proliferation, invasion, and metastasis. Moreover, lncRNA ROR modulates tamoxifen resistance through miR-205 regulation and engages other miRNAs critical to EMT, collectively advancing metastasis and cancer progression (McGuire et al., 2015; Zhang H. Y. et al., 2017).

In breast cancer, the long non-coding RNA LINC00922 enhances EMT through methylation of NKD2, supporting invasive and metastatic phenotypes (Wang Y. et al., 2021). Overexpressed in breast cancer tissues, LINC00922 is closely linked to tumor aggressiveness and poor prognosis. It recruits DNA methyltransferases (DNMT1, DNMT3A, DNMT3B) to the NKD2 promoter, facilitating NKD2 suppression via methylation (Wang Y. et al., 2021). As NKD2 inhibits the Wnt pathway, its reduced expression activates Wnt signaling, driving EMT, cell proliferation, invasion, and migration (Wang HB. et al., 2019). Furthermore, elevated LINC00922 levels correlate with higher histological grade, TNM stage, tumor size, lymph node involvement, negative progesterone receptor status, and features of breast cancer stem cells, notably self-renewal and multidrug resistance (Lou et al., 2018).

In addition, studies have shown that DLX6-AS1 and lncRNA ZEB2-AS1 play crucial roles in regulating the EMT process in breast cancer cells. DLX6-AS1 inhibits EMT by silencing the expression of specific lncRNAs, while the ectopic expression of TTN-AS1 promotes EMT by upregulating EMT-related genes. SOX21-AS1, on the other hand, influences the EMT process in breast cancer by suppressing the expression of vimentin and N-cadherin, two key markers of mesenchymal phenotype (Su et al., 2022).

#### 5.2.2 Regulation of key signaling pathways involved in EMT

In breast cancer, lncRNA-ANCR suppresses invasion and metastasis by promoting phosphorylation and degradation of EZH2. LncRNA-ANCR enhances the interaction between CDK1 and EZH2, leading to phosphorylation of EZH2 at Thr-345 and Thr-487, which promotes its ubiquitination and degradation (Li Z. et al., 2017). EZH2, a critical epigenetic regulator, undergoes various post-translational modifications (PTMs) that affect its stability (Yan et al., 2017; Kim and Roberts, 2016). lncRNA-ANCR expression is typically reduced in breast cancer tissues and cell lines compared to normal tissues. ANCR knockdown induces EMT in MCF10A cells and promotes migration and invasion, while ectopic ANCR expression inhibits these processes in breast cancer cells. In a nude mouse model, ANCR overexpression significantly reduced the tumor-forming ability of MDA-MB-231 breast cancer cells and prevented lung metastasis (Li Z. et al., 2017). These findings suggest that lncRNA-ANCR inhibits breast cancer invasion and metastasis by destabilizing EZH2, highlighting lncRNA-ANCR as a potential therapeutic target.

LncRNA-ATB promotes transforming growth factor-β (TGF-β)-mediated EMT and drug resistance by competitively binding to miR-200c (Yuan et al., 2014). In breast cancer, TGF-β stimulation upregulates lncRNA-ATB, which binds miR-200c, releasing suppression on EMT regulators like ZEB1 and ZNF217 and promoting their expression. This mechanism enhances breast cancer cell invasiveness and metastasis (Shi et al., 2015). Elevated lncRNA-ATB expression is linked to poor prognosis, with significantly higher levels in tumor tissues than in normal breast tissues (Li J. et al., 2017). Overexpression of lncRNA-ATB in breast cancer cell lines induces EMT marker changes, including E-cadherin downregulation and N-cadherin and vimentin upregulation, correlating with increased invasiveness. Additionally, lncRNA-ATB overexpression is associated with trastuzumab resistance, impacting breast cancer cell sensitivity to this metastasis-inhibiting drug (Garcia et al., 2013).

Additionally, lncRNA-ATB is closely associated with cancer stem cell characteristics, promoting tumor self-renewal and multidrug resistance (Chen et al., 2019b). It has also been identified as a direct target of the TGF-β/Smad signaling pathway (Yuan et al., 2014).

In TNBC, lncRNA ZFAS1 inhibits EMT and tumor progression by targeting STAT3, a transcription factor critical to cancer cell proliferation, survival, and invasion (Sharma et al., 2021; Wang et al., 2020). Studies have shown a negative correlation between ZFAS1 expression and STAT3 activity, with reduced ZFAS1 levels corresponding to increased protein levels of STAT3 and its phosphorylated form in TNBC cells. ZFAS1 interacts with STAT3, inhibiting the activation of downstream signaling pathways and reducing the expression of EMT-related transcription factors, such as Slug and ZEB1 (Sharma et al., 2021). These factors drive EMT by downregulating epithelial markers (e.g., E-cadherin) and upregulating mesenchymal markers (e.g., N-cadherin and vimentin), promoting cancer cell invasion and metastasis (Shibue and Weinberg, 2017). Furthermore, ZFAS1 also suppresses TNBC cell proliferation and colony formation by regulating CDK inhibitors p21 and p27, with silencing of ZFAS1 enhancing these processes while its overexpression inhibits them (Sharma et al., 2021).

NME1 (nucleoside diphosphate kinase 1) regulates the expression of EMT-related genes, influencing cellular epithelial or mesenchymal states (Prunier et al., 2023). Research by Guo et al. demonstrated that LINC00261, which is downregulated in breast cancer, inhibits cell proliferation and migration via the NME1-EMT pathway (Guo et al., 2020). LINC00261 binds NME1 mRNA, preventing its degradation and increases NME1 protein levels. Upregulated NME1 modulates EMT-related genes, impacting the cell’s epithelial or mesenchymal status (Huna et al., 2021).

Overexpression of LINC00261 effectively inhibits breast cancer cell proliferation and migration, while silencing LINC00261 is sufficient to induce tumor formation in breast cancer. Additionally, LINC00261 suppresses breast cancer cell invasion and metastasis by regulating the expression of EMT markers such as E-cadherin and N-cadherin (Guo et al., 2020).

In breast cancer, long non-coding RNA NEAT1 promotes cell proliferation, EMT, and metastasis by modulating specific molecular pathways (Park et al., 2021). Liu et al. demonstrated that NEAT1 accelerates breast cancer progression by regulating the miR-410-3p/CCND1 axis (Liu et al., 2020). Acting as a molecular “sponge,” NEAT1 binds miR-410-3p, thereby relieving its suppression of CCND1 and increasing CCND1 protein levels. Overexpressed CCND1, a key cell cycle regulator, is strongly linked to enhanced breast cancer cell proliferation and invasion (Montalto and De Amicis, 2020). NEAT1 also drives invasion and metastasis by modulating EMT markers, including upregulation of N-cadherin and vimentin and downregulation of E-cadherin (Park et al., 2021). Additionally, NEAT1 interacts with transcription factors and chromatin remodeling complexes to regulate EMT-related gene expression, positioning NEAT1 as a potential therapeutic target in breast cancer (Knutsen et al., 2022).

### 5.3 Key circRNAs regulating EMT

Circular RNAs regulate key transcription factors and signaling pathways in EMT, acting as competitive endogenous RNAs or through direct targeting. These mechanisms influence breast cancer cell migration, invasion, and metastasis. Abnormal circRNA expression offers potential as therapeutic targets or diagnostic biomarkers in breast cancer. Below is a summary of key circRNAs involved in EMT regulation in breast cancer.

#### 5.3.1 Direct inhibition of EMT transcription factors (EMT-TFs) or regulation of cytoskeletal components (epithelial and mesenchymal genes)

Hsa_circ_0008039 is a novel circular RNA that is upregulated in breast cancer tissues. Studies have shown that it promotes breast cancer cell proliferation and migration by functioning as a competitive endogenous RNA (ceRNA) for miR-432-5p, thereby regulating the expression of E2F3(182). Specifically, hsa_circ_0008039 acts as a ceRNA, sequestering miR-432-5p and relieving its inhibitory effect on E2F3, leading to increased E2F3 protein levels. E2F3 is a key regulator of the cell cycle, and its upregulation accelerates cell cycle progression and enhances cell proliferation (Chong et al., 2009) Moreover, the overexpression of E2F3 is associated with increased migration of breast cancer cells, likely through mechanisms involving cell adhesion and cytoskeletal reorganization (Jusino et al., 2021). Thus, the hsa_circ_0008039/miR-432-5p/E2F3 regulatory axis plays a promotive role in breast cancer progression and may represent a potential therapeutic target for future breast cancer treatments.

In breast cancer, circANKS1B promotes tumor invasion and metastasis through complex regulatory mechanisms. As a molecular “sponge,” circANKS1B sequesters miR-148a-3p and miR-152-3p, relieving their suppression of the transcription factor USF1 and leading to increased USF1 expression (Zeng et al., 2018). This mechanism regulates cytoskeletal components by downregulating epithelial markers like E-cadherin and upregulating mesenchymal markers such as vimentin (Zeng et al., 2018).

USF1, as a transcription factor, further enhances the transcription of TGF-β1, activating the TGF-β1/Smad pathway, a key driver of EMT and breast cancer cell invasion and metastasis (Casalino et al., 2023). In this pathway, TGF-β1 binds its receptors, activating downstream Smad proteins, including R-Smad and Co-Smad (e.g., SMAD4). These Smad complexes translocate to the nucleus, where they regulate target genes involved in cell proliferation, differentiation, migration, and apoptosis (Miyazono, 2000).

In breast cancer, activation of the TGF-β1/Smad signaling pathway is linked to tumor invasion and metastasis, with circANKS1B enhancing this pathway by regulating USF1 and TGF-β1 expression (Zeng et al., 2018). Additionally, miR-148a-3p and miR-152-3p act as tumor suppressors across various cancers, inhibiting tumor growth by targeting genes involved in cancer progression. For instance, in prostate cancer, miR-148a-3p and miR-152-3p suppress KLF4 expression, limiting tumor cell growth (Zeng et al., 2018).

#### 5.3.2 Regulation of key signaling pathways involved in EMT

CircANKS1B regulates USF1 expression by sponging miR-148a-3p and miR-152-3p, thereby upregulating TGF-β1 transcription and activating the TGF-β1/Smad signaling pathway (Zeng et al., 2018). This pathway induces EMT, promoting breast cancer cell invasion and metastasis. In breast cancer, TGF-β signaling regulates downstream transcription factors through both Smad-dependent and Smad-independent mechanisms, including Snail, ZEB, and bHLH family members, all critical for EMT regulation (Pastushenko et al., 2021).

In addition, circRNAs regulate molecules and signaling pathways, including the PI3K/AKT/mTOR pathway is essential for breast cancer cell proliferation, survival, migration, apoptosis, glucose metabolism, and DNA repair (Miricescu et al., 2020). The PI3K/AKT/mTOR pathway influences EMT by regulating the activity of transcription factors. For instance, the activation of AKT upregulates the expression of EMT-inducing factors such as Snail and Slug, which suppress epithelial markers like E-cadherin and induce the expression of mesenchymal markers such as N-cadherin and Vimentin (Huang et al., 2021). As a key node, mTOR activation promotes cell proliferation, survival, and metabolic reprogramming and is closely linked to EMT. mTOR also modulates microRNA expression to regulate EMT-related gene translation (Liu et al., 2011). The mTOR signaling pathway consists of two complexes, mTORC1 and mTORC2, which play pivotal roles in controlling cell growth and survival. mTORC1 is activated via the PI3K/AKT pathway, while mTORC2 further promotes AKT activation by directly phosphorylating it at Ser473 (Miricescu et al., 2020). Additionally, the HER family receptors (including EGFR, HER2, HER3, and HER4) also contribute to the development and progression of breast cancer, particularly in HER2-positive breast cancer, by activating the PI3K/AKT/mTOR pathway (Heldin et al., 2012).

## 6 Potential clinical applications of ncRNAs in breast cancer

Growing evidence indicates that ncRNAs play a pivotal role in BC development and progression, particularly in the EMT process. Circulating tumor cells (CTCs) with EMT and CSC markers have been detected in blood samples from BC patients (Luo et al., 2024). Uncovering the molecular mechanisms regulating EMT is therefore essential for developing effective strategies to treat and prevent BC metastasis. For example, secreted TGFβ2 has been suggested as a potential biomarker and therapeutic target for TNBC (Luo et al., 2024). Additionally, ncRNAs are closely associated with clinical and pathological features of BC, such as molecular subtypes, tumor grade, lymph node metastasis, and distant metastasis, making them valuable tools for expression profile screening in tumor and adjacent tissues. Research, for instance, has shown that circACTN4 expression is significantly upregulated in BC tissues compared to normal breast tissues and is closely correlated with tumor stage in BC patients (Wang X. et al., 2021).

NcRNAs have emerged as potential biomarkers and therapeutic targets for EMT in breast cancer. For instance, studies have shown that in a TGF-β-induced EMT model, lncATB promotes breast cancer cell invasion and metastasis by acting as a ceRNA for miR-200c, thereby regulating Twist1 expression (Li et al., 2018). Wu et al. identified miR-410 through real-time PCR and Western blotting as a direct inhibitor of growth and metastasis in ER-positive breast cancer, with ERLIN2 highlighted as a potential biomarker for early screening and diagnosis (Wu et al., 2018). Additionally, various miRNAs, including miR-449a, miR-206, and miR-26, function as tumor suppressors or oncogenes in breast cancer pathogenesis (Wu et al., 2018). Research has also shown that BCSCs overexpressing ZEB1 exhibit strong chemotherapy resistance. BCSCs are regulated by signaling pathways such as TGF-β, Wnt, Notch, Hedgehog (HH), and NF-κB, all having critical roles in treatment resistance, cancer recurrence, and metastasis (Luo et al., 2015). Further investigation is essential to understand how these pathways regulate BCSCs (Wang et al., 2022). Identifying these miRNAs could provide fundamental insights for targeting key signaling pathways in BCSCs for therapeutic intervention.

Current research is investigating immunotherapies targeting BCSCs, including CAR-T cell therapy, immune checkpoint inhibitors, and vaccines. A primary challenge is selectively targeting BCSCs without affecting MaSCs or normal non-cancerous cells (Khan et al., 2021). One clinical study in TNBC developed a HA-LSL/siTGF-β nanoparticle platform that reshaped the tumor microenvironment. When combined with anti-PD-L1 therapy, it elicited a robust anti-tumor immune response and significantly improved therapeutic efficacy (Yang et al., 2023).

Additionally, drugs targeting other pathways, such as the CDK 4/6 inhibitor Palbociclib, are in clinical trials with endocrine therapy and aromatase inhibitors, showing promising results (NCT02738866, NCT03709082, NCT02491983) (Khan et al., 2021). Gene-editing technologies like CRISPR/Cas9 are also showing potential in breast cancer treatment. For instance, tBSA/Cas9-PAR2 nanoparticles designed to edit the PAR2 gene have effectively inhibited breast cancer metastasis (Fu et al., 2023). Detecting and regulating ncRNAs involved in breast cancer EMT offers substantial potential for clinical application. Future developments in early screening biomarkers and precision therapeutic targets are expected to improve breast cancer prognosis, prevention, and treatment.

## 7 Discussion

Breast cancer remains a significant global health challenge due to its high incidence and mortality rates. Prognosis is closely linked to the tumor’s invasiveness and metastatic potential, with EMT recognized as a critical driver of invasion and metastasis (Bray et al., 2024). Emerging research highlights the pivotal role of ncRNAs, including miRNAs, lncRNAs, and circRNAs, in regulating EMT. These ncRNAs not only facilitate tumor cell invasion and metastasis through their influence on EMT-related transcription factors and signaling pathways but also play roles in interactions between BCSCs and tumor cells, immune evasion, and treatment resistance. The role of ncRNAs in EMT offers insights into mechanisms underlying breast cancer invasion and metastasis. For example, miRNAs regulate EMT-related protein synthesis by targeting specific mRNAs; the miR-200 family suppresses EMT by targeting ZEB1 and ZEB2, while miR-205 reduces EMT by downregulating ZEB2, which is associated with improved prognosis in breast cancer (Gregory et al., 2008a). LncRNAs also modulate EMT through various mechanisms. For instance, lncRNA ZEB2-AS1 promotes proliferation, metastasis, and EMT in TNBC by epigenetically activating ZEB2. CircRNAs similarly influence EMT, such as circANKS1B, which regulates the TGF-β1/Smad signaling pathway to drive invasion and metastasis in TNBC. Additionally, lncRNAs like HOTAIR and MALAT1 enhance EMT and metastasis through interactions with chromatin remodeling complexes. CircRNAs, by acting as miRNA sponges or interacting with proteins, further regulate EMT-related gene expression (Memczak et al., 2013).

The precise roles of ncRNAs in breast cancer, particularly their cross-regulation and collective impact on EMT, remain insufficiently explored. Furthermore, the influence of ncRNAs on modulating EMT characteristics in BCSCs is unclear, presenting significant research opportunities. A deeper understanding of these mechanisms—especially regarding EMT, the tumor microenvironment, immune editing, drug resistance, and radiosensitivity—could open new avenues for breast cancer treatment. Given the strong correlation between ncRNAs and clinical outcomes in breast cancer, ncRNAs show considerable promise as biomarkers for tumor diagnosis, subtype classification, therapy, and prognosis, potentially surpassing other liquid biopsy methods like circulating tumor cells.

Despite advances in understanding the roles of ncRNAs in breast cancer EMT, therapeutic applications remain challenging due to issues with stability, delivery efficiency, and tissue-specific expression *in vivo*. Additionally, the effects of tumor heterogeneity and patient variability on ncRNA function require further investigation. Future research should examine how these factors interact with ncRNAs and collectively influence breast cancer biology, offering new perspectives and strategies for disease prevention and treatment.
